# Seasonal Variation in Mycorrhizal Community of Different Cerrado Phytophysiomies

**DOI:** 10.3389/fmicb.2020.576764

**Published:** 2020-10-26

**Authors:** Wagner Gonçalves Vieira Junior, Jadson Belem de Moura, Rodrigo Fernandes de Souza, Ana Paula Maciel Braga, Diogo Jânio de Carvalho Matos, Gustavo Henrique Mendes Brito, José Mateus dos Santos, Rodrigo Martins Moreira, Sandro Dutra e Silva

**Affiliations:** ^1^Soil Research Group, Ecology and Dynamics of Organic Matter, Evangelical College of Goianésia, Goianésia, Brazil; ^2^Agricultural Microbiology Program, Faculty of Agricultural and Veterinary Sciences, Paulista State University Júlio de Mesquita filho, Jaboticabal, Brazil; ^3^Graduate Program in Natural Resources of the Cerrado, State University of Goiás, Anápolis, Brazil; ^4^Graduate Program in Genetics and Plant Breeding, Federal University of Goiás, Goiânia, Brazil; ^5^Department of Environmental Engineering, Federal University of Rondônia, Ji-Paraná, Brazil; ^6^Laboratory of Cerrado Environmental History, State University of Goiás, University Center of Anápolis, UniEVANGELICA, Anápolis, Brazil

**Keywords:** savannas, water deficit, environment, vesicular-arbuscular mycorrhiza, Fungi (arbuscular mycorrhizal fungi)

## Abstract

**Purpose:** Understanding the dynamics of mycorrhizal fungi in the Cerrado is fundamental for the adoption of conservation practices and for understanding the resilience of this biome in relation to long periods of drought. Thus, this work aimed to verify the dynamics of the mycorrhizal population in five phytophysiognomies of the Cerrado biome.

**Methods:** The samples were taken from the Chapada dos Veadeiros National Park, a permanent preservation with native Cerrado vegetation without any anthropic influence. The five main phytophysiognomies of the Cerrado biome were chosen: the Campo Limpo, Campo Sujo, Cerrado Strictu Sensu, Cerradão, and Veredas. Rhizospherical soil samples were collected in both the wet and dry seasons. Spore density, mycorrhizal colonization rate, easily extractable glomalin, and associated mycorrhizal fungi genera were identified.

**Results:** The values of spore density, mycorrhizal colonization rate, and glomalin were higher in the samples performed during the dry season compared to the samples performed in the rainy season. The same behavior was observed when comparing the different phytophysionomies.

**Conclusion:** Mycorrhizal activity is higher in dry periods when compared to rainy periods. There is no specificity of genera of arbuscular mycorrhizal fungi within the Cerrado phytophysiognomies.

## Introduction

Brazil is the largest country in Latin America and one of the world’s leading food producers. Much of its territorial extension, about 35% of the Brazilian territory, is covered by a type of vegetation classified as Cerrado (Batalha, 2011). The Cerrado is the second largest Brazilian biome, extending over an area of 2,045,064 km^2^, covering eight states of Central Brazil: Minas Gerais, Goiás, Tocantins, Bahia, Maranhão, Mato Grosso, Mato Grosso do Sul, Piauí, and Distrito Federal (Hunke et al., 2015). It includes three of the largest hydrographic basins in South America, with regular rainfall indexes that provide it with great biodiversity. Being behind only the area occupied by the Amazon Rain Forest, but in reality, the Cerrado is widely considered the last agricultural frontier of the Americas, as it is the last area where agricultural expansion is still possible. (Braz et al., 2004; Klink and Machado, 2005; Dutra e Silva, 2020).

The Cerrado is one of the largest biodiversities on the planet, because it is a transitional biome that connects other important biomes such as the Amazon, the Caatinga, the Atlantic Coast Forest, the Pantanal, and the Bolivian Chacos (Taber et al., 1997; Klink and Machado, 2005). The Cerrado is also considered one of the hotspots for the conservation of global biodiversity. The predominant soil class in this biome is Oxisols, which are deep soils of low natural fertility, acids, with intense weathering, rich in iron oxides, and aluminum, but deficient in phosphorus (dos Santos et al., 2013). Another important feature of the Cerrado is the climate, defined as humid tropical, with two well-defined seasons, a dry winter and humid summer. The dry season is usually between April and September and the wet season between October and March (Cardoso et al., 2015).

This seasonality has a direct influence on the mycorrhizal fungi population of the Cerrado soils. According to Pirozynski (1981), the association of mycorrhizal with superior vegetables started its evolution in species that are only found in tropical regions. Because they absorb water through plants, which increases their resistance to water deficit, mycorrhizal fungi plays a fundamental role in the Cerrado biome, especially in the dry season (Augé, 2001). Surveys conducted of different soil types of this biome shows that arbuscular mycorrhizal fungi (AMF) are associated with a large number of native plants. This encompassing grasses, legumes, and tree species such as the pequi (*Caryocar brasiliense*) and the buriti (*Mauritia flexuosa*; de Miranda, 2008). Of the 79 species of AMF found in Brazilian biomes, 67% were identified in soils of the Cerrado biome, being closely linked to the edaphoclimatic conditions of the region (Moreira and Siqueira, 2006; Moura and Cabral, 2019; Moura et al., 2019).

Understanding the dynamics of mycorrhizal fungi in the Cerrado is fundamental for the adoption of conservation practices to preserve this biome and understanding its resilience with the help of the microbial community in relation to long periods of drought. The objective of this work is to verify the dynamics of the mycorrhizal population in five phytophysiognomies of the Cerrado biome during the seasons.

## Materials and Methods

**Figure 1:**
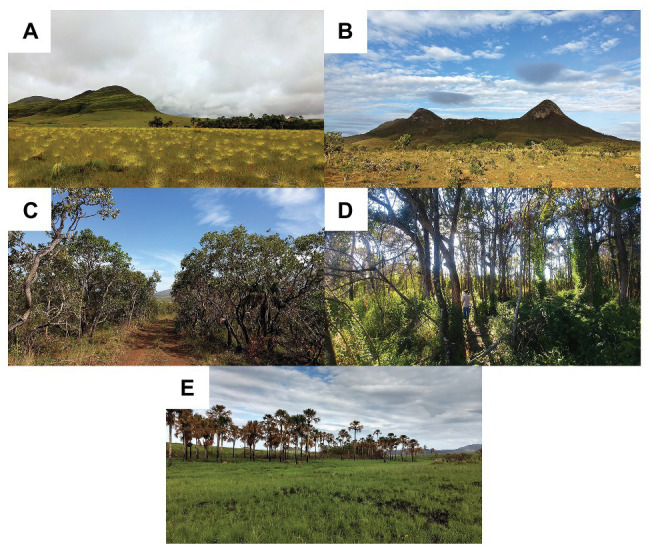
The five main phytophysiognomies of the Cerrado biome: Campo Limpo **(A)**, Campo Sujo **(B)**, Cerrado Strictu Sensu **(C)**, Cerradão **(D)**, and Veredas **(E)**.

The samples were collected in the Chapada dos Veadeiros National Park, a permanent preservation region with native Cerrado areas without anthropic influence. The five main phytophysiognomies of the Cerrado biome were chosen according to the classification of Batalha (2011), Campo Limpo, Campo Sujo, Cerrado Strictu Sensu, Cerradão, and Veredas (Figure 1). Phytophysiognomies are classified by areas that have the same type of vegetation, such as savannas, forests, fields, and prairies (Batalha, 2011).

Four different periods were chosen to collect samples, two in rainy season (November 2017 and February 2018) and two in the dry season (June and September 2018). Each time, 12 replicates of root and rhizospherical soil samples of each phytophysiognomy were collected; each sample was composed by mixing five subsamples collected randomly at a depth of 0–20 cm. The floristic survey of the species of phytophysiognomies was not carried out.

The analyses were carried out at the Laboratory of Agricultural Microbiology of the Evangelical College of Goianésia. AMF spores were extracted from 50cm^3^ rhizospherical soil by wet sieving technique (Gerdemann and Nicolson, 1963) followed by centrifugation in water and sucrose solution 50%. The spores were separated according to their phenotypic characteristics, such as color, size, and shape, composing the different morphotypes, under stereoscopic binocular magnifying glass.

To determine the percentage of colonization, the roots were clarified and stained with 0.05% trypan blue in lactoglycerol (Phillips and Hayman, 1970), and the evaluation of colonization performed under stereoscopic microscope, following the technique of quadrant intersection (Giovannetti and Mosse, 1980).

The extraction of easily extractable glomalin (EEG) was obtained by weighing 1 g of soil mixed in 8 ml of sodium citrate at 20 mM (pH 7.0). Afterward, it was autoclaved for 30 min at 121°C. At the end, centrifugation was performed for 20 min at 5000 rpm (Wright and Upadhyaya, 1996, 1999). For the quantification of extractable glomalin from the soil, the Bradford methods modified by Wright and Upadhyaya (1996) were used, using bovine sero-albumin as standard protein, and a spectrophotometer at a reading of 595 nm.

For the identification of AMF genera from morphological characteristics, the spores were separated according to their morphotypes and mounted on slides with pure polyvinyl-lacto-glycerol (PVLG) and PVLG mixed with Melzer (1:1 v/v). To support the identification work, original articles of the description of the species provided on the website of the “International Culture Collection of Arbuscular and Vesicular-Arbuscular Mycorrhizal Fungi” (INVAM, 2018).

The data were submitted to variance analysis by the Assistat program (Silva, 2008), and canonical correspondence statistics were performed by past software (Hammer, 2018).

## Results

**Figure 2:**
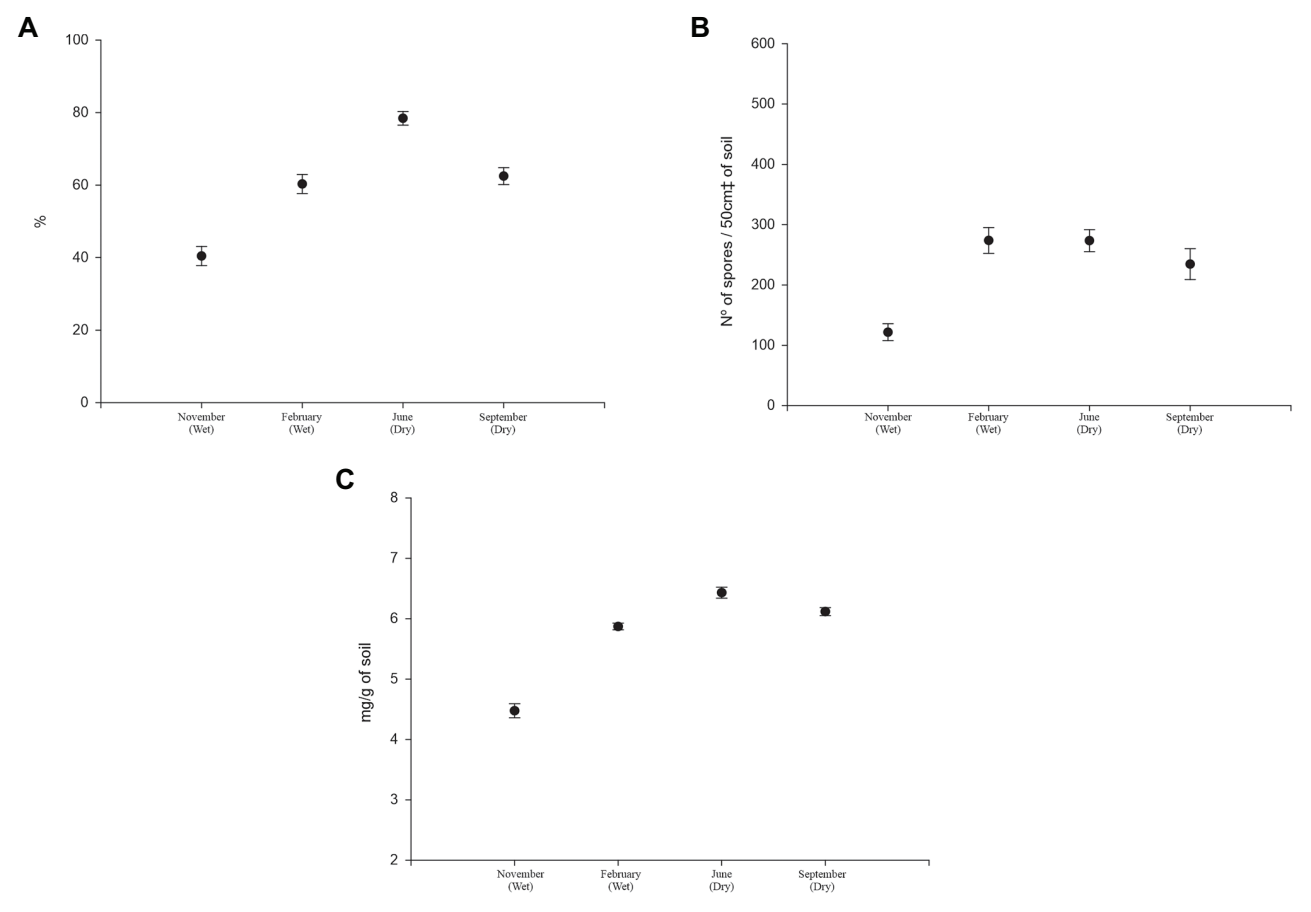
**(A)** Mycorrhizal colonization rate (%), **(B)** spore density (number of spores/50cm^3^ of soil), and **(C)** glomalin easily extractable (mg/g of soil) of soils under Cerrado in rainy seasons (first and second) and dry season (third and fourth).

The seasonal distribution in the Cerrado interfered with the dynamics of mycorrhizal fungi associated with their different phytophysiognomies. Figure 2 shows the values of mycorrhizal colonization rate, spore density, and glomalin in four samplings over the course of a year, regardless of phytophysiognomies.

The three parameters have similar behavior. The mycorrhizal colonization rate recorded was 40.43% in the first sampling, which occurred in November at the beginning of the rainy season; 60.29% in the second sampling, which occurred in March, at the end of the rainy season; 78.43% in June, at the beginning of the dry season, and 62.47% in September, at the end of the dry season (Figure 2A).

The mean density of spores present in rhizosphere at the first collection was 121.58 spores/50 cm^3^ of soil. In the second sampling, the value was 273.8 spores/50 cm^3^ of soil. The third sampling registered 273.4 spores/50 cm^3^ of soil, and 234.5 spores/50 cm^3^ of soil in the last sampling was verified (Figure 2B).

The values of glomalin easily extractable were 4.47 mg g^−1^, 5.87 mg g^−1^, 6.43 mg g^−1^, and 6.11 mg g^−1^ of soil in the 1st, 2nd, 3rd, and 4th collections, respectively (Figure 2C).

**Figure 3:**
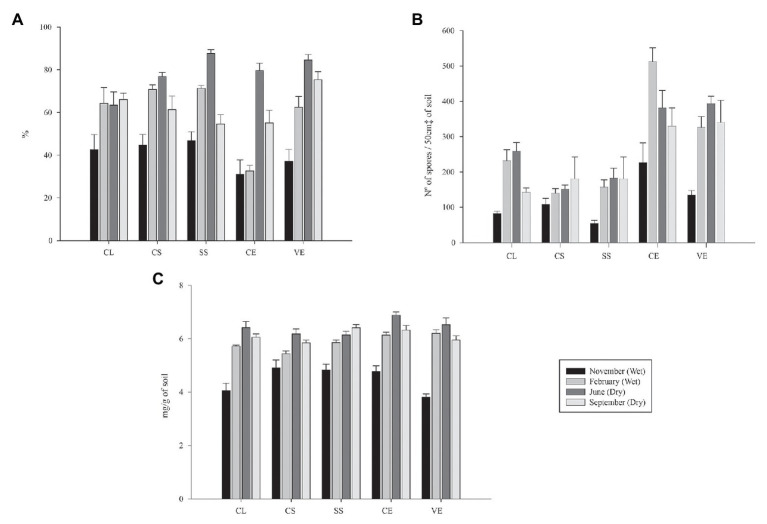
Mycorrhizal colonization rate **(A)**, spore density **(B)**, and glomalin easily extractable **(C)** from soils from the Cerrado during the rainy (first and fourth) and dry seasons (second and thirrd). CL, Campo Limpo; CS, Campo Sujo; SS, Strictu Sensu; CE, Cerradão; and VE, Veredas.

By separately analyzing the phytophysiognomies Campo Limpo (CL), Campo Sujo (CS), Cerrado Strictu Sensu (SS), Cerradão (CE), and Veredas (VE), it is possible to verify a similar behavior of mycorrhizal fungi in relation to the season (Figure 3).

While analyzing the mycorrhizal colonization rate data (Figure 3A), all phytophysiognomies presented similar behavior, where the values in the first sampling were low, following an increase in the second and third sampling, and a drop in the fourth sampling compared to the third.

Regarding the values of density of spores in the soil, phytophysiognomies followed the same behavior as the mycorrhizal colonization rate, except for the samples gathered at Campo Sujo, which demonstrated an increase in the number of spores from the first to the fourth sampling (Figure 3B). When observing the values of glomalin easily extractable, the values increased until the third sampling, and decrease in the fourth sampling when compared to the third, except for the values observed in the Strictu Sensu phytophysiognomy, which the values increased at each sampled time (Figure 3C).

Table 1 shows the genera identified in the soil of the five phytophysiognomies studied in the four collections performed.

**Table 1:** Genera of arbuscular mycorrhizal fungi (AMF) identified in rhizosphere of different Cerrado phytophysiognomies in the dry and rainy season, in which (+) corresponds to the presence of the Genus.

Genera	*Acaulospora*				*Claroideoglomus*				*Diversispora*				*Sclerocystis*			
Sampling	Nov (wet)	Feb (wet)	Jun (dry)	Sep (dry)	Nov (wet)	Feb (wet)	Jun (dry)	Sep (dry)	Nov (wet)	Feb (wet)	Jun (dry)	Sep (dry)	Nov (wet)	Feb (wet)	Jun (dry)	Sep (dry)
Campo Limpo	+	+	+		+	+	+	+	+	+	+	+				+
Campo Sujo	+	+		+	+	+	+	+	+	+	+	+				+
Strictu Sensu	+	+		+	+			+	+	+	+	+				+
Cerradão	+	+	+		+	+	+	+	+	+	+	+				+
Veredas	+	+	+	+	+	+	+	+	+	+	+	+				
**Genera**	***Glomus***				***Funneliformis***				***Gigaspora***				***Scrobiculata***			
**Sampling**	**Nov (wet)**	**Feb (wet)**	**Jun (dry)**	**Sep (dry)**	**Nov (wet)**	**Feb (wet)**	**Jun (dry)**	**Sep (dry)**	**Nov (wet)**	**Feb (wet)**	**Jun (dry)**	**Sep (dry)**	**Nov (wet)**	**Feb (wet)**	**Jun (dry)**	**Sep (dry)**
Campo Limpo	+	+	+	+	+	+		+	+	+	+					
Campo Sujo	+	+	+	+	+	+		+	+	+		+				
Strictu Sensu	+	+	+	+	+	+		+	+	+	+					
Cerradão	+	+	+	+	+	+	+	+	+	+	+	+	+	+		
Veredas	+	+	+	+	+	+		+	+	+	+	+	+			

The genera *Glomus* and *Diversispora* were the only ones found in all phytophysiognomies and samples. The genera *Acaulospora*, *Claroideoglomus*, *Funneliformis*, and *Gigaspora* were also identified at a high frequency in phytophysiognomies. *Sclerocystis* and *Scrobiculata* were the least identified genera in the investigated soils.

The genera *Acaulospora*, *Claroidoglomus*, *Diversispora*, and *Gigaspora* were abundant in the samples extracted during both rainy seasons. The genus *Scrobiculata* and *Funneliformis* were present only in the second sampling.

Likewise, the genera *Acaulospora*, *Claroidoglomus*, *Diversispora*, and *Gigaspora* were found to be abundant in the samples taken during the dry season. The *Slcerocystis* genre was identified only in the last sampling.

**Figure 4:**
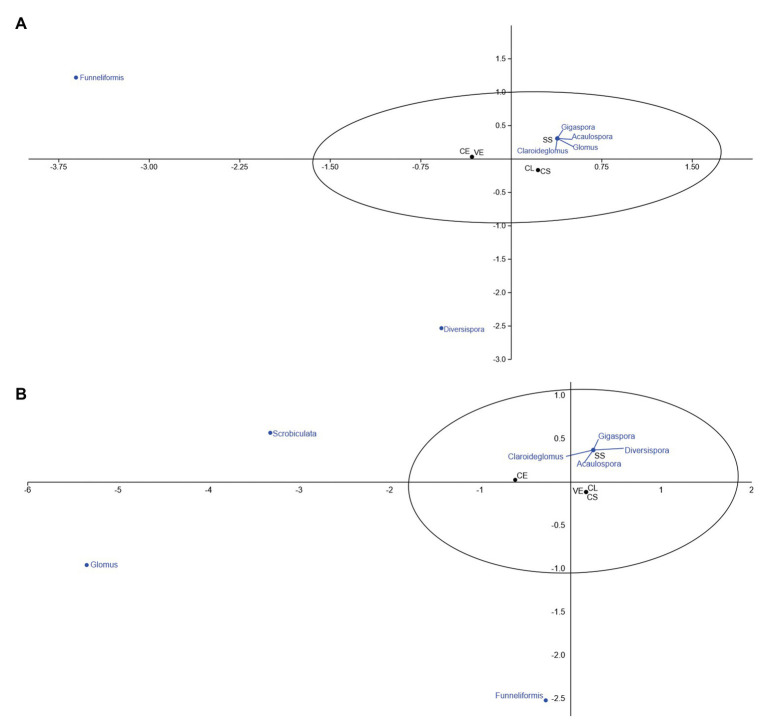
Canonical correspondence of mycorrhizal fungi associated with different Cerrado phytophysiognomies in the rainy season. **(A)** November and **(B)** February. CL, Campo Limpo; CS, Campo Sujo; SS, Strictu Sensu; CE, Cerradão; and VE, Veredas.

Canonical correspondence analysis aims to correlate the incidence of AMF genera identified with the frequency found in each phytophysiognomies. Figure 4 correlates the frequency of fungal genera associated with phytophysiognomies in the first collection.

**Figure 5:**
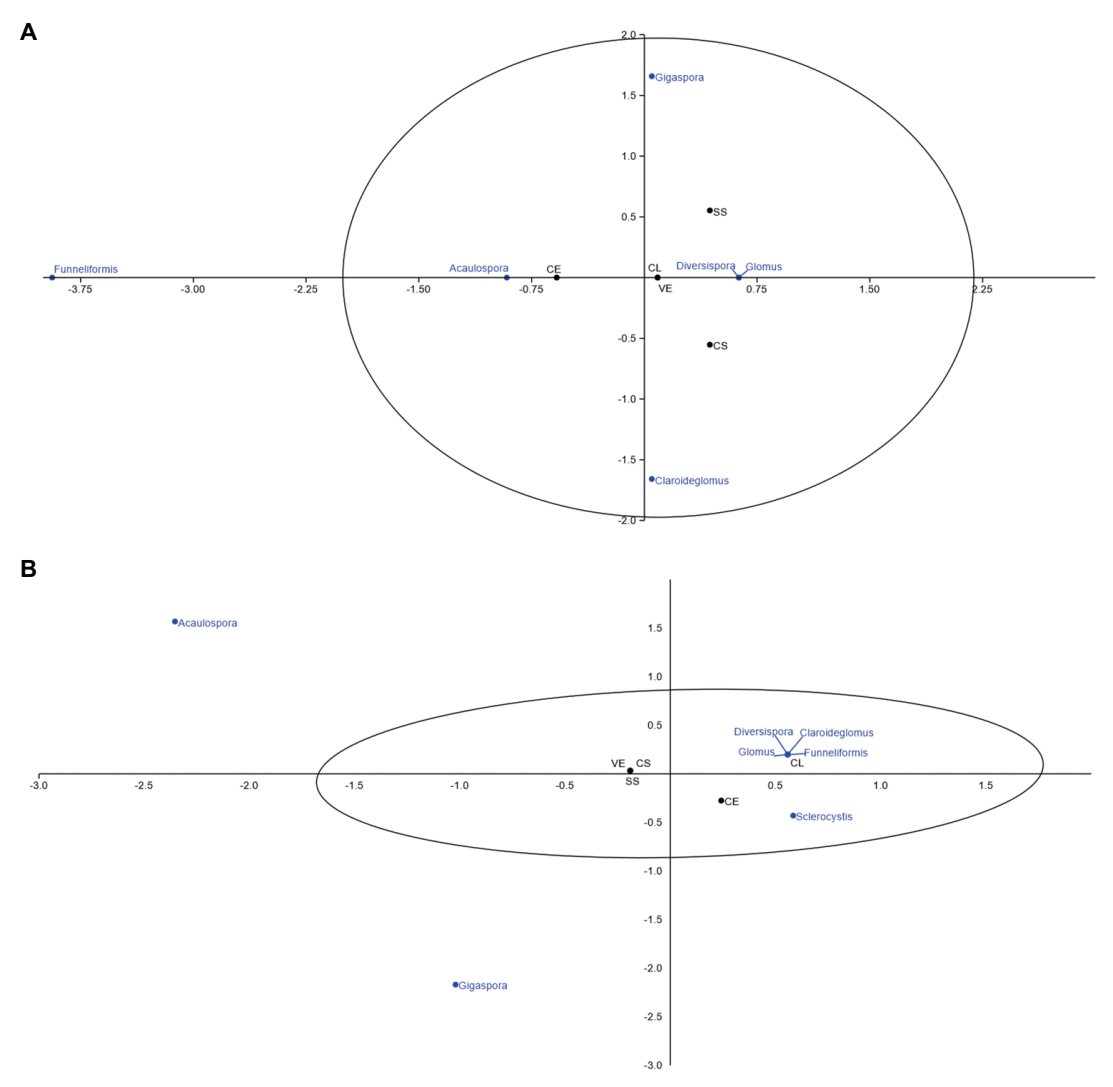
Canonical correspondence of mycorrhizal fungi associated with different Cerrado phytophysiognomies in the dry season. **(A)** June and **(B)** September. CL, Campo Limpo; CS, Campo Sujo; SS, Strictu Sensu; CE, Cerradão; and VE, Veredas.

Of all identified genera, *Diversispora* and *Scrobiculata* are not normally associated with Cerrado phytophysiognomies. The genera *Funneliformis*, *Gigaspora*, *Claroideoglomus*, *Acaulospora*, and *Glomus* have a high affinity with the Strictu Sensu Cerrado type (Figure 4A). In the second collection (Figure 5), the genera *Scrobiculata* and *Claroideoglomus* have a low correlation with phytophysiognomies, and *Funneliformis*, *Gigaspora*, *Diversispora*, *Acaulospora*, and *Glomus* are commonly associated with the rhizosphere of the phytophysiognomies studied.

Figure 5 presents the canonical correspondence analyses of the frequency of AMF associated with rhizosphere of five Cerrado phytophysiognomies in the dry season.

Only the genus *Funneliformis* was identified at the beginning of the dry season, (Figure 5A). The genera *Acaulospora* and *Sclerocystis* were unexpectedly found in the samples investigated at the end of the dry season (Figure 5B). The other genera showed greater affinity with Campo Limpo during the dry season.

## Discussion

The climate in the Cerrado Biome is defined as humid tropical, with two well-defined seasons, a dry winter and a humid summer (da Silva et al., 2008; Cardoso et al., 2015). With aspects that can be interpreted as adaptations to dry environments, the Cerrado’s landscape displays trees and shrubs with tortuous trunks; deep roots to improve efficiency in water absorption; thick, corky bark to reduce evapotranspiration loss; hardened, coriaceous, and bright surface leaves; and production of flowers and sprouts in the middle of the dry season, which confirms the vegetation of this biome were adapted to these climatic conditions (Goedert, 1989; Beuchle et al., 2015; Ferri, 2017).

The Cerrado biome is an environment that naturally offers adverse abiotic conditions for plant growth and development. With low phosphate levels and limited water regime, plants depend directly on the performance of mycorrhizal fungi to resist such conditions, attributing to the association between fungi and plants, which is an important resilience factor to stressful situations (Thomazini, 1974; Porcel and Ruiz-Lozano, 2004; Hunke et al., 2015; Moura et al., 2017).

According to Pirozynski (1981), the mycorrhizal association with vascular vegetables would have begun its evolution in the tropics, and there are species that are even found only in these regions. Today, its presence is currently reported in different regions of the planet, regardless of the region’s climate (Khan, 1993; Vestberg, 1995; Aliasgharzadeh et al., 2001; Muthukumar et al., 2004; Gehring and Connell, 2006; Moura et al., 2017).

A significant number of surveys carried out in different types of Cerrado soil show that AMF are associated with a large number of plants native to the biome, encompassing grasses, legumes, and tree species, such as the pequi (*Caryocar brasiliense*) and the buriti (*Mauritia flexuosa*; de Miranda, 2008).

The importance of AMF in water absorption and aid to tolerance to long periods of drought is already known. This group of organisms plays a fundamental role for the maintenance of this ecosystem, considering that the Cerrado is an ecosystem, where its climate pattern presents a long semester of drought (Moura and Cabral, 2019). The values of spore density, mycorrhizal colonization rate, and easily extractable glomalin are excellent indicators of the action of AMF in the biome studied during seasonal variation.

Figures 4, 5 demonstrate the expected behavior of mycorrhizal fungi in ecosystems with these seasonal characteristics. During the dry season, the colonization rate tends to increase. When plants are submitted to water stress conditions, they received from fungi present in the soil to increase the absorption rates of watered nutrients (Morte et al., 2000; Al-Karaki et al., 2004). This explains the highest values in the third and fourth samples, collected during the drought season.

The Cerrado Biome has great biodiversity and there are differences between its vegetations that allow the classification in five different phytophysiognomies with similar plant characteristics. The Cerrado type Campo Limpo contains the predominance of creeping grasses. The Campo Sujo contains the predominance of shrubby plant species. The Strictu Sensu has as predominant vegetation tortuous trees, with thick bark and coriaceous leaves. The Cerradão is a woodland territory, with dense forest and large trees. Finally, the Vereda type of vegetation presents a predominance of the buriti, a kind of Palmaceae, established in lowland areas and regions close to bodies of water (Klink and Machado, 2005; Batalha, 2011; Ferri, 2017).

These characteristics explain the higher values of spore density in the Cerradão phytophysiognomy (Figure 4B), with high plant density of different species, promoting a rich rhizosphere, and consequently, a greater biodiversity of edaphic organisms.

There was no specificity of the genera associated with the rhizosphere of the phytophysiognomies investigated. The genera *Glomus*, *Diversispora*, and *Gigaspora* were found in virtually all types of Cerrado. These genera are commonly found in tropical regions. Studies developed by de Miranda (2008) found these genera in agroecosystems of Cerrado. Moura et al. (2017) verified the presence of these genera in sugarcane under Cerrado soil, and other studies also verified the presence of these genera, indicating low specificity to plant species, and may colonize most of the vegetables of this biome (Casagrande, 1985; Vieira, 2001; Lacerda et al., 2011; Angelini et al., 2012; Ferreira et al., 2012).

Glomalin is a glycoprotein produced by AMF and released into the soil acting on soil aggregation and structuring (Hammer and Rillig, 2011). The glomalin values found in the present study are close to those found by Santos (2016) from the Cerrado soil, which ranged from 2.1 to 4.4 mg g^−1^. Fokom et al. (2012), when they evaluated areas of consortium with peanut, corn, banana, and cassava crops, they also found values of 6.51 mg g^−1^. In forested areas, these authors found more expressive values reaching 10.56 mg/g of soil.

The production of glomalin varies according to the species of mycorrhizal fungi found in colonization. Wright and Upadhyaya (1999) found difference in glomalin production according to the AMF species studied. In cultivation in the culture medium, *Gigaspora rosea* and *Gigaspora gigantea* had higher productivity than *Glomus intraradices* and *Glomus etunicatum* (Wright and Upadhyaya, 1996).

In general, glomalin values were higher in the dry season than in the rainy season (Figure 3C). The production of this protein by the fungus is a response to environmental stresses, such as drought and salinity (Hammer and Rillig, 2011). Soon after the beginning of the rainy season, the glomalin values fall again in all sampled areas, indicating a decrease in the activity of the fungus.

Water stress is one of the triggers for the mycorrhizal association between the fungus and the plant, and glomalin levels can be excellent indicators of mycorrhizal activity in the soil. Glomalin values are in tune with the density of spores found in the same areas. Easily extractable glomalin values are correlated with the time of year, considering the climate. Protein values peaked in the rainy season, in almost all samples of sample 3, the values were more expressive compared to data that were collected with higher water availability in which they obtained the lowest concentration. Cogo (2016) also found in a typical Dystrophic Red Latosol, higher glomalin levels in dry seasons. This can occur due to the death of hyphae and their decomposition in the soil. Silva et al. (2013) found a larger amount of glomalin in summer periods compared to winter at the Atlantic Coast forest soils.

## Conclusion

Mycorrhizal activity is higher in dry seasons when compared to the rainy seasons. There is no specificity of genera of AMF with the Cerrado phytophysiognomies investigated. This serves as evidence that the dynamics of the mycorrhizal population present in the soils of the Cerrado biome are more influenced by the season than by the phytophysiognomies studied.

## Data Availability Statement

The raw data supporting the conclusions of this article will be made available by the authors, without undue reservation.

## Author Contributions

WV: responsible for writing. JM: project advisor. RS: responsible for statistical analysis. AB: responsible for glomalin analyzes. DM: responsible for the analysis of mycorrhizal fungi. GB: responsible for maps. JS: responsible for the analysis of mycorrhizal fungi. RM: responsible for maps. SD and JM: postdoctoral supervisor. All authors contributed to the article and approved the submitted version.

### Conflict of Interest

The authors declare that the research was conducted in the absence of any commercial or financial relationships that could be construed as a potential conflict of interest.
